# Correction: Hypothemycin, a fungal natural product, identifies therapeutic targets in *Trypanosoma brucei*

**DOI:** 10.7554/eLife.01214

**Published:** 2013-07-23

**Authors:** Mari Nishino, Jonathan W Choy, Nathan N Gushwa, Juan A Oses-Prieto, Kyriacos Koupparis, Alma L Burlingame, Adam R Renslo, James H McKerrow, Jack Taunton

Nishino M, Choy JW, Gushwa NN, Oses-Prieto JA, Koupparis K, Burlingame AL, Renslo AR, McKerrow JH, Taunton J. 2013. Hypothemycin, a fungal natural product, identifies therapeutic targets in *Trypanosoma brucei*. *eLife* **2**:e00712. doi: http://dx.doi.org/10.7554/eLife.00712. Published 09 July 2013

Hypothemycin was incorrectly spelled ‘hypothemicin’ in the article title.

The article has been corrected accordingly.

